# Can adaptive expertise, reflective practice, and activity theory help achieve systems-based practice and collective competence?

**Published:** 2019-07-24

**Authors:** Angela Orsino, Stella Ng

**Affiliations:** 1 Holland Bloorview Kids Rehabilitation Hospital, Ontario, Canada; 2 Centre for Faculty Development, University of Toronto, Ontario, Canada; 3 Li Ka Shing International Healthcare Education Centre, St. Michael’s Hospital, Ontario, Canada

## Abstract

Physicians must function as integral members of the complex social systems in which they work to support the health of their patients; competency-based education frameworks describe this function of physicians in terms of systems- based practice, advocacy, and collaboration. Yet education for these social competencies continues to present challenges, perhaps because medical education has tended to focus less on social systems and more on traditional healthcare systems. In this paper, we use a clinical example from the discipline of Developmental Pediatrics, that of early identification of autism spectrum disorder (ASD), as an illustration of a socially complex zone of practice necessitating systems-based practice. We first explore this practice context through the framings of collective competence and activity theory to represent the complex practices and systems involved in identifying ASD. We then align these framings of the practice context and complexity with two bodies of education theory, adaptive expertise and reflective practice. We argue that these approaches to education will prepare learners to be more aware of and responsive to the dynamic needs of the complex and intersecting systems in which they will practice.

## Systems-based practice in medical education

Physicians are expected to function as integral members of the complex social systems in which they work to support the health of their patients. Reflecting this common practice need, competency frameworks outline roles or profiles to guide education efforts. In the U.S., the Accreditation Council for Graduate Medical Education Outcome Project outlines Systems-Based Practice as a core competency. This competency requires residents to “demonstrate an awareness of and responsiveness to the larger context and system of health care, as well as the ability to call effectively on other resources in the system to provide optimal health care.”^1^ Similarly, in Canada, The Royal College of Physicians and Surgeons of Canada outline Health Advocate and Collaborator as expected competencies, and call for physicians to work as part of a broader system or collective in their daily practice.^2^ Recent scholarship on health advocacy has argued convincingly for a systems mind-set and collective effort in how advocacy is taught and practiced.^3^

Although the importance of systems-based practice in optimal clinical care is clearly stated, educating and assessing medical trainees in this area continues to be a challenge.^4,5^ In this paper, we use a clinical example from the discipline of Developmental Pediatrics, that of early identification of autism spectrum disorder (ASD), as an illustration of a socially complex zone of practice necessitating systems-based practice. We explore this practice context through the framings of collective competence and activity theory to represent the complex practices and systems involved in identifying ASD. We then align these framings of the practice context and complexity with two bodies of education theory, adaptive expertise and reflective practice. We argue that these approaches to education will prepare learners to be more aware of and responsive to the dynamic needs of the complex systems in which they will practice.

## A case for a collectivist approach

In the field of Developmental Pediatrics, early identification and intervention for children with ASD is prioritized to optimize future developmental outcomes. However, despite efforts to provide primary care providers with knowledge of ASD and screening tools,^6-8^ many children continue to be identified later than ideal,^9^ delaying connection to vital supports and interventions.

Optimal care for children with special needs necessitates integrated care across medical, health care and educational systems.^10-12^ A toddler with ASD flags may receive medical check-ups from their primary care provider, as well as be assessed by a speech and language or occupational therapist in the community. Some children also attend childcare placements prior to entering school. One can thus envision these children having access to a distributed team of individuals working in different systems, dispersed across time and settings, each encounter offering an opportunity for identification of developmental concerns and prompting of further assessment. Intersectoral collaboration proves challenging given the need to integrate multiple individuals, interdisciplinary teams, systems, policies and agencies, each with their own set of knowledge and norms.^13-15^

One reason for the continued challenges may be that the various paediatric-related professions are trained within a paradigm of individual competence; that is, individuals are graduated, licensed to practice, and regulated by their own professional college according to their own scope of practice.^16^ Through these processes, clinicians are socialized to work in silos or as individuals on relatively stable and co-located teams. Given this context, clinicians may be challenged to share goals, to identify and use shared communication pathways, and to be aware of how their practices intersect with those of others located elsewhere. Clinicians and other practitioners, distributed across multiple systems, may or may not see themselves as part of a unified “team,” with the child at the center of this team.

Addressing these challenges calls for a paradigm shift in health professions education. It requires educators to acknowledge that currently existing training models may reinforce gaps in practice between independent practitioners in different disciplines, and that a more collectivist viewpoint is necessary. To bridge such gaps, educational models that target the development of collective competence, to complement the individual competence of those involved in a distributed team, are necessary.

Collective competence is a distributed capacity of a system, not easily reducible to an individual; it is a competence that is dynamic, not a static state that is achieved and maintained; it is strongly tied to context.^16^ Given the complexity of children’s needs, educational models that acknowledge collective competence are necessary, but currently lacking, across pediatric-focused disciplines. Furthermore, actual practical approaches that could foster collective competence ideals remain elusive.

## Understanding the systems to inform education

Our first step in better understanding how to realize collective competence in health professions education was to draw upon theory that allows one to describe systems and their essential elements. Several approaches offer such descriptive capacity, including critical discourse analysis, actor network theory and activity theory. For our descriptive analysis in this paper, we chose to use activity theory with its focus on understanding human action and learning, accounting for not just an individual but also his/her social and cultural context.^17^ Activity theory can be used to map different systems involved in a social situation (such as the various medical, health, and education training programs involved in educating professionals involved in child development). Using this theory, an activity system describes how an individual subject engages in action, using tools, towards a particular object, emphasizing the importance of sociocultural context. Elements that interact with and influence subjects achieving objects and eventual outcomes within an activity system are summarized in Table 1 below. Activity theory can help unpack the many interacting activity systems involved in a social situation, such as that of early ASD identification, to promote communication and alignment of outcomes between subsystems.

**Table 1:** Activity system elements in two representative medical educational systems

Activity System Elements	Developmental Pediatrics subspecialty residency	General Pediatrics residency
**Subjects** (the individuals working within the system)	Subspecialty residents Developmental Pediatric faculty Families/children	Pediatric residents Academic and community pediatricians Developmental Pediatricians Families/children
**Object** (immediate purpose/objectives of the action)	Development of knowledge and skills to recognize early ASD flags and diagnose appropriately	Development of knowledge and skills to detect early child development concerns and ASD flags through developmental surveillance and use of developmental screening tools^11^ Diagnosis of ASD
**Outcome** (the product sought by the system)	Early diagnosis of ASD Early intervention Improved developmental outcomes	Early referral for ASD diagnostic assessment Early diagnosis of ASD Early intervention Improved developmental outcomes
**Rules** (explicit/implicit norms or expectations)	Developmental pediatricians/residents diagnose ASD	Pediatricians/residents perform developmental surveillance, early detection of developmental delays and may diagnose ASD Pediatricians as gatekeepers to developmental pediatricians Barriers to screening^6^ and diagnosis^18^ Debate around evidence for screening tools^19^ Lack of uniform screening protocol across North America
**Artifacts** (conceptual or material tools used by people within system)	ASD diagnostic tools Diagnostic criteria	Developmental surveillance guidelines/tools ASD screening guidelines^11^ and tools Diagnostic criteria ± diagnostic tools
**Community** (environment/people surrounding subjects)	Hospital or community clinics Childhood educators Child health professionals/therapists	Hospital or community clinics Childhood educators Child health professionals/therapists
**Division of labour** (roles of subjects within system)	Developmental pediatricians/residents conduct diagnostic assessment Developmental pediatricians teach residents about ASD diagnosis and assessments	Pediatricians teach developmental surveillance, screening tools and diagnosis to residents Developmental pediatricians teach residents about ASD diagnosis and assessments

Using activity theory, we are able to think about educational planning in the context of systems, and thus shift our focus from the individual toward the collective involved in ASD identification. We acknowledge that many different groups of health professions learners may be a part of a child’s distributed team. As an attempt to begin to explore this issue, we selected just two salient postgraduate medical education systems involved in training around child development (Pediatrics and Developmental Pediatrics residency programs) to explore using activity theory. An abbreviated example of this process is shown in Table 1. Such mapping efforts allow individuals in a system to see beyond their individual paths to consider how they intersect with others within and across systems. Further, this mapping offers a language for systems-based collectivist educational approaches.

Initial mapping of these systems provided us with a glimpse into the complexity associated with attending to the collective in developing actual educational approaches. We outlined how elements seemingly shared between systems may, on the surface, appear obvious or the same, while actually holding different and even conflicting understandings between systems. For example, we noted variability in both explicit and implicit rules and division of labour, which can possibly impact clarity of individual roles and intended educational and practice objectives within and across systems. For example, the pivotal role of paediatricians in ASD diagnosis is clearly endorsed,^11^ yet several factors may lead to a shift in emphasis of paediatricians’ roles and misalignment of outcomes, complicating the realization of collective competence. These factors include overlapping roles with developmental pediatricians, tensions in the system (e.g., barriers to use of screening tools^6^ and communicating diagnoses^18^), and lack of consistently recommended ASD screening protocols across North America.

## Preparing learners for practice in dynamic systems

Any educational initiative aimed at developing collective competence would have to raise awareness that one’s own individual learning and practice is situated in a broader, complex intersection of interacting systems. In our particular clinical context this encompasses medical, rehabilitation, and other health disciplines/professions, childcare and education systems which are all subject to sociocultural influences. A collectivist approach to medical education would also require adaptation to the dynamic nature of these systems. Activity theory clearly details the constant shifting of tensions within systems related to misaligned outcomes, and suggests that innovation occurs when these misalignments productively create new objects.^20^ Teaching with a collectivist goal in mind could contribute to training physicians toward more effective systems-based practice.

More specifically, in the clinical context of early ASD identification, education interventions across health professions that provide individual knowledge about ASD and skill in using screening tools are important but insufficient.^7,8^ We argue they would be bolstered by an awareness of and capacity to engage with the collective and dynamic aspects of practice. To provide this awareness and capacity, educational interventions may need to aim to prepare adaptive experts and reflective practitioners who can integrate individual knowledge and skill with systems awareness.^21,22^

The development and implementation of educational curricula informed by the theories of adaptive expertise and reflective practice could help prepare learners to practice more effectively in complex systems. For example, a curriculum informed by principles of adaptive expertise would be able to train learners to appropriately use their knowledge in both routine and non-routine problem-solving situations to provide solutions to clinical problems. Inclusion of integrated biomedical teaching around clinical signs, diagnostic criteria, and assessment/screening methods for ASD would support problem-solving and learning during non-routine cases. One could argue that some of the unexpected systems issues arising in everyday ASD care, which lead to a need to advocate or create system work-arounds, represent a non- routine case.^23,24^ It has also been shown that learners are able to develop a deeper understanding of clinical concepts when they engage in active learning activities (e.g., exploring contrasting cases) prior to learning from a didactic lecture.^25^ One can thus envision a curriculum that includes prerequisite active learning exercises for learners in which they are exposed to various presentations of developmental delays in children before more direct instruction is provided. Assessing learners’ competence in both efficient and innovative dimensions of adaptive expertise using dynamic assessments would be necessary to evaluate both what learners know as well as how well they are prepared to use new knowledge to innovate.^21^ Mylopoulos and Woods describe one such type of assessment that combines questions that require the application of acquired knowledge alongside questions that require students to learn new knowledge in order to successfully problem solve.^24^

Relatedly, curricula centered on reflective practice would focus on an overarching framing of practice as inherently uncertain. The marker of professional practice, according to reflective practice, is to be able to recognize and respond well to situations for which there are no definitive solutions. Practitioners enact reflective practice in response to what Schön termed indeterminate zones of practice, which are ambiguous, unstable, unique or value conflicted aspects of work.^22^ We argue that developmental pediatrics is rife with these zones (as are many other practice areas). Teaching approaches informed by reflective practice would include similar methods to those described above in the adaptive expertise section. Additionally, faculty would emphasize the inherent ambiguity of practice and help socialize students into a tolerance or even appreciation of ambiguity, in-the-moment problem-solving, and thoughtful innovation. Further, critical reflection, which can be defined as reflective activity with a specific focus on challenging status quo, power dynamics, and taken-for-granted but unhelpful assumptions, could be taught and assessed. Approaches for teaching critical reflection have been detailed in other papers^26,27^ and include presenting stories that disrupt assumptions, for example, presenting images that depict singular, dominant representations of disability, and asking learners what messages the image sends, who benefits from such representations, and who is harmed.^28^

## Summary

The authors acknowledge that we have only scratched the surface of this issue through our limited exploration of two educational activity systems; however, our aim is to kindle further thought and discussion in this area. Successful coordinated care and management of children with complex needs, such as those presenting with early signs of ASD, requires physicians who are capable in systems-based practice and related competencies. Developmental pediatrics practice goals, including navigating systems and advocating with and for patients and families, align well with medical education approaches that target the development of collective competence. Activity theory allowed us to describe systems and deconstruct activities according to their individual elements. The mapping of sociocultural and material elements of representative systems highlighted important aspects to consider in systems-based practice and collective competence goals. Two bodies of education theories, adaptive expertise and reflective practice, spanning separate yet overlapping paradigms of education may offer potential teaching and learning strategies that can help achieve the goals of systems-based practice and collective competence. Though an early exploration, beginning to bring together four vast bodies of theory in this paper allowed us to consider and conceptualize ways forward for teaching toward a goal shared by all four: effective practice in complex systems.
